# Microbial reassembly-driven multi-niche functional compensation alleviates uranium stress in rapeseed

**DOI:** 10.3389/fmicb.2026.1860545

**Published:** 2026-07-23

**Authors:** Di Guo, Chang Liu, Meifeng Wang, Zhirui Niu, Yanlong Chen

**Affiliations:** 1 School of Petroleum and Environment Engineering, Yan’an University, Yan’an, Shaanxi, China; 2 School of Life Sciences, Northwestern Polytechnical University, Xi’an, Shaanxi, China

**Keywords:** assembly, functional compensation, microbial community, rapeseed, soil sterilization, uranium

## Abstract

**Background:**

Uranium (U) pollution harms soil and plants. It is still unclear how microbes help plants survive uranium stress in different root and leaf environments.

**Objectives:**

In this study, We examined how soil microbial reassembly affects rapeseed growth and the microbes living in soil, roots, and leaves under uranium stress, and explored the functional compensation mechanisms involved.

**Methods:**

A pot experiment was conducted with rapeseed grown in three soil backgrounds (original, sterilized, sterilized-inoculated) under control and 150 mg⋅kg^–1^ uranium stress. Plant growth, uranium content, and 16S rRNA sequences from four niches (bulk soil, rhizosphere, roots, leaves) were analyzed.

**Results:**

Uranium predominantly accumulated in roots, with limited translocation to shoots. Uranium stress increased chlorophyll content but still induced chlorosis. Ecological niche shaped microbial community structure more strongly than soil treatment or uranium stress. Even though the microbial community did not fully return to its original state, it became enriched with uranium-tolerant, growth-promoting bacteria (e.g., *Mesorhizobium*, *Streptomyces*, *Castellaniella*). The reassembled community was associated with alleviated photosynthetic damage and improved phenotype under uranium stress.

**Conclusion:**

Niche differentiation primarily drives community assembly, and maximizing function does not require complete structural restoration. The naturally enriched multifunctional consortia under uranium stress provide a promising basis for plant–microbe combined remediation.

## 1 Introduction

Uranium (U), a critical raw material for the nuclear industry, can enter into the soil and water environment during mining, milling, and nuclear waste disposal. It poses both chemical toxicity and radiological hazards, potentially leading to kidney injury, DNA damage, and carcinogenic risks through the food chain (Srivastava et al., 2019; Zhang et al., 2022). In some uranium mining areas and surrounding farmlands in China, soil uranium concentrations have far exceeded background levels, threatening ecological security and sustainable agricultural development (Zhao et al., 2023). Consequently, cost-effective and efficient remediation technologies for uranium-contaminated soil are urgently needed.

In soil, uranium predominantly exists as the uranyl ion (UO_2_^2+^) and its hydrolyzed complexes. It is readily adsorbed and retained by plant root cell walls and vacuoles, with minimal translocation to aboveground tissues (Ratnikov et al., 2019). Uranium also triggers harmful reactive oxygen species (ROS) in plant cells, damaging chloroplasts and reducing photosynthetic rate (Chen et al., 2022). Rhizosphere microbe-assisted phytoremediation has attracted increasing attention, as microorganisms can alter uranium speciation and bioavailability or alleviate uranium toxicity to plants through phytohormone production, induced systemic resistance, and antioxidant enzyme secretion (Cui et al., 2023). However, existing studies have largely focused on the isolation and inoculation of single functional strains (Kaur et al., 2025; Liang et al., 2025), whereas the response of complex microbial communities, their niche differentiation patterns, and the link between community restoration and plant protective functions under uranium stress remain poorly understood at the community level.

Compared original, sterilized, and sterilized-inoculated soils to understand how microbial structure relates to function. By comparing original, sterilized, and sterilized-inoculated soils, it is possible to elucidate the recovery capacity of microbial communities, niche assembly rules, and mechanisms of functional redundancy and compensation under stress (Hassi et al., 2023). However, this approach has rarely been applied to uranium-contaminated soil–plant systems, particularly in conjunction with high-throughput sequencing and comparative analysis across multiple ecological niches (bulk soil, rhizosphere, root endosphere, and leaf phyllosphere).

Therefore, using rapeseed, we investigated (i) the stepwise microbial recruitment from bulk soil to leaf phyllosphere under uranium stress; (ii) how sterilized-inoculated treatment regulates this process; and (iii) the effects of soil treatment and uranium stress on community assembly, structural recovery, functional compensation, and enrichment of key uranium-tolerant taxa across four niches. These findings will provide a theoretical foundation and scientific reference for understanding plant-microbe interactions in uranium-contaminated environments and for the targeted manipulation of soil microbiomes.

## 2 Materials and methods

### 2.1 Experimental soil, plant cultivation, and sample collection

Soil was collected from farmland surrounding Northwestern Polytechnical University at 0–15 cm depth. After removing stones and plant roots, the soil was air-dried, passed through a 40-mesh sieve, and autoclaved (121 °C, 20 min, twice with a 24 h interval) for sterilization; a portion was set aside for inoculum preparation. Basic physicochemical properties are provided in Supplementary Table 1. The test plant was rapeseed (Brassica napus cv. Nanma Aiyou 188). Plants were grown in a climate-controlled chamber (70% relative humidity, 16 h light/28 °C and 8 h dark/20 °C) and supplemented every 2 days with sterile deionized water and half-strength Hoagland nutrient solution. After 6 weeks, samples were collected from four compartments: bulk soil (>2 cm away from roots, collected after gently shaking to remove roots); rhizosphere soil (tightly attached to roots was collected with a brush); root endophytes (roots rinsed with sterile water, blotted dry); and leaf phyllosphere (upper mature leaves surface-sterilized with 70% ethanol for 2 min, rinsed three times with sterile water). Each sample was divided into two portions: one snap-frozen in liquid nitrogen and stored at −80 °C for microbial diversity analysis, the other for uranium determination.

### 2.2 Soil sterilization, inoculation, and experimental design

The soil suspension for inoculation was prepared by mixing 20 g of fresh soil with 190 mL of autoclaved deionized water and stirring for 2 min. The soil suspension (10^–1^ dilution) was collected, and then serially diluted to 10^–6^ and 10^–9^; each dilution was added to sterile soil bags. qPCR analysis showed that microbial abundance was similar across dilutions during the 8-week incubation period. The experiment included three soil treatments: original soil (unsterilized), autoclaved sterilized soil (121 °C, 20 min, twice with 24 h interval), and sterilized-inoculated soil (sterilized soil inoculated with the microbial suspension). Six treatment groups were established with three replicates each, totaling 18 pots: A_U (original soil + 150 mg kg^–1^ U), A (original soil control), B_U (sterilized-inoculated soil + 150 mg kg^–1^ U), B (sterilized-inoculated soil control), C_U (sterilized soil + 150 mg kg^–1^ U), and C (sterilized soil control). Sampling niches are abbreviated as RS (rhizosphere soil), S (bulk soil), R (roots), and L (leaves). Uranium was added as uranyl nitrate hexahydrate [UO_2_(NO_3_)_2_⋅6H_2_O]. A complete list of treatment codes and descriptions is provided in Supplementary Table 2.

### 2.3 Plant physiological measurements

Roots and shoots were oven-dried at 105 °C for 30 min to deactivate enzymes, then at 75 °C to constant weight for dry biomass determination (g plant^–1^). Photosynthetic pigments were extracted from fresh rapeseed leaves by cutting into ∼0.2 cm pieces, and 0.2 g was placed in a 25 mL volumetric flask. Then, 0.5 mL of pure acetone and 15 mL of 80% acetone were added, and the flask was sealed and incubated in the dark at room temperature overnight with intermittent shaking. After leaf tissues turned completely white, the volume was brought to 25 mL with 80% acetone. The supernatant was collected after centrifugation, and absorbance was measured at 663 nm (chlorophyll a) and 645 nm (chlorophyll b) using a spectrophotometer. Chlorophyll content was calculated using standard equations (Jeffrey and Humphrey, 1975).

### 2.4 Determination of uranium content in rapeseed tissues

Fresh roots were first immersed in 25 mM Na2EDTA solution (with shaking for 15 min) to remove surface-adsorbed uranium, then rinsed three times with deionized water before drying and grinding. Dried samples were digested using a microwave digestion system with an HNO_3_-H_2_O_2_ mixture. After cooling, digested samples were diluted, and total uranium concentration was measured using Inductively Coupled Plasma Mass Spectrometry (ICP-MS, PerkinElmer, United States). Throughout the measurement process, standard reference material (GBW07603) was used for quality control, and blank and parallel samples were prepared to ensure data accuracy. Three biological replicates were analyzed.

### 2.5 Microbial community structure analysis

Total genomic DNA was extracted from bulk soil, rhizosphere, root, and leaf samples using the DNeasy PowerSoil Pro Kit (Qiagen); root and leaf samples were ground in liquid nitrogen prior to extraction. The V3–V4 region of the 16S rRNA gene was amplified with primers 338F (5’-AACMGGATTAGATACCCKG-3’) and 806R (5’-ACGTCATCCCCACCTTCC-3’). Paired-end sequencing (2 × 300 bp) on an Illumina MiSeq platform yielded 1,265,794 raw reads. Primers were trimmed with cutadapt, and DADA2 was used for quality filtering, denoising, merging, and chimera removal to generate ASVs. ASVs were clustered into Operational Taxonomic Unit (OTUs) at 97% similarity using VSEARCH. Taxonomy was assigned via QIIME2’s classify-sklearn against Greengenes 13.8, and a phylogenetic tree built with the MAFFT–FastTree pipeline. The OTU table was rarefied to 95% of the minimum sample depth. Negative controls yielded negligible reads. Raw sequences were deposited in NCBI SRA. Alpha diversity (observed OTU richness, Shannon index) and rarefaction curves were computed. Beta diversity was assessed using Bray–Curtis and weighted/unweighted UniFrac distances and visualized by PCoA. Community composition was summarized from phylum to genus, and biomarker taxa identified with LEfSe. Co-occurrence networks were constructed from the OTU matrix to calculate topological indices and identify potential keystone species.

### 2.6 Statistical analysis

OTU tables were rarefied to 95% of the minimum sequencing depth using QIIME2. Rank-abundance curves and Venn diagrams assessed species richness and shared/unique taxa. PCoA based on Bray-Curtis distances visualized community structure dissimilarities. Co-occurrence networks were built from the rarefied OTU matrix after filtering low-abundance OTUs (present in <20% of samples). Pairwise Spearman correlations were calculated using SparCC, with significance determined by random matrix theory (| ρ| > 0.6, *P* < 0.01, FDR-corrected). Networks were generated with igraph, and the top 100 most abundant nodes visualized with ggraph. Network properties (node/edge counts, positive/negative edge ratio, modularity, average clustering coefficient) were computed. Module hubs (Zi ≥ 2.5) and connectors (*P*i ≥ 0.62) were identified based on within- and among-module connectivity. These co-occurrence patterns do not imply direct ecological interactions or functional control. Network centrality (e.g., Zi, Pi) was used only to identify highly connected taxa, not to infer keystone species or causal ecological roles.

## 3 Results

### 3.1 Plant growth and uranium accumulation

**FIGURE 1:**
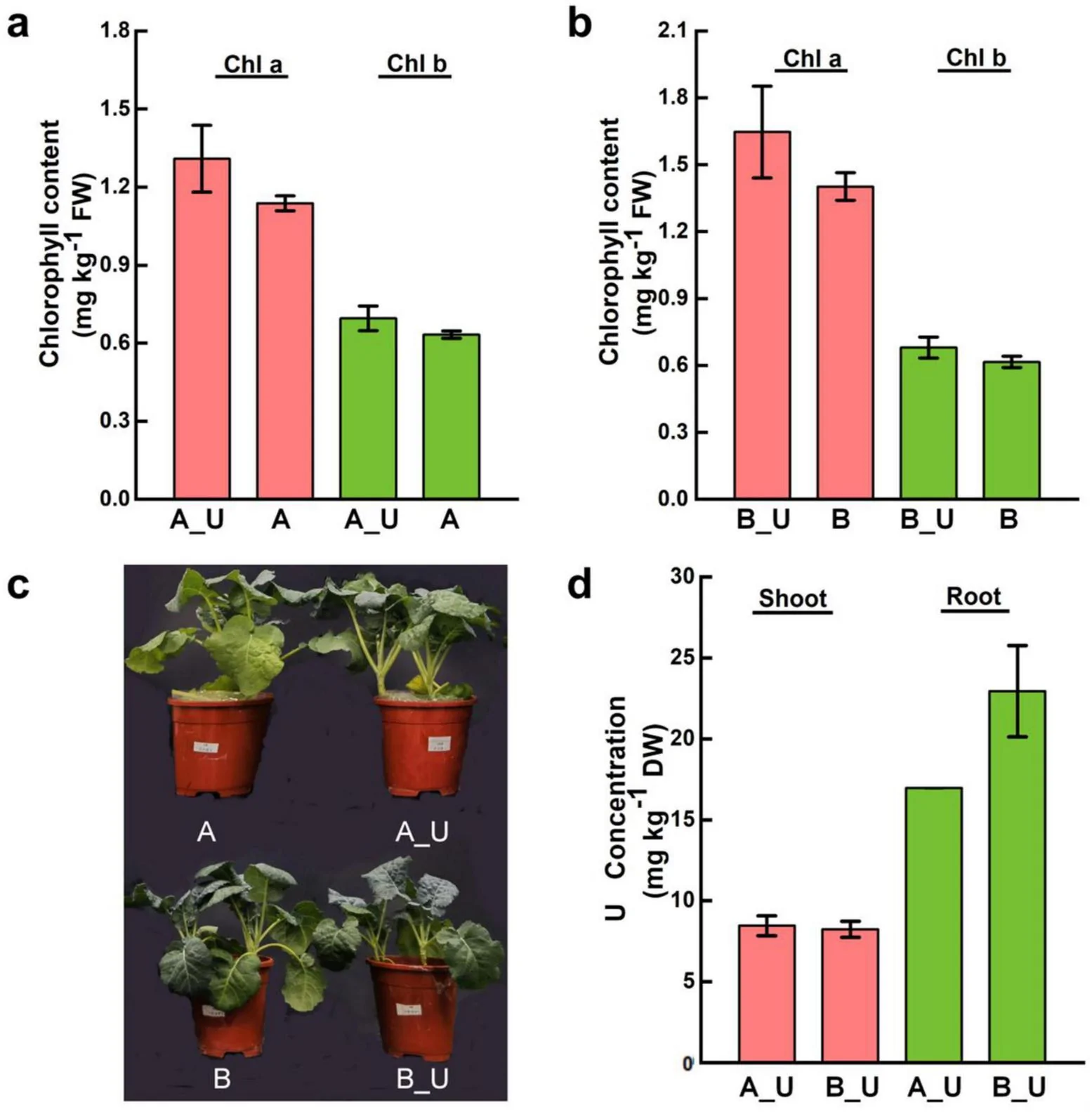
Rapeseed growth, chlorophyll content, and uranium accumulation under different soil treatments. **(a)** Chlorophyll a (pink bars) and chlorophyll b (green bars) in leaves of rapeseed grown in original soil without (A) or with (A_U) uranium (150 mg kg^−1^ soil). Y-axis: milligrams of chlorophyll per kilogram fresh leaf weight. Bars are mean ± standard deviation (n = 3); **(b)** Same as **(a)** but for sterilized-inoculated soil (reinoculated soil) without (B) or with (B_U) uranium. **(c)** Representative photographs of whole plants from each treatment group. A: original soil control (normal green leaves). A_U: original soil + uranium (note chlorosis and leaf curling). B: reinoculated soil control (green leaves, shorter stature). B_U: reinoculated soil + uranium (dark green, expanded leaves, no chlorosis). **(d)** Uranium concentration in roots (green bars) and shoots (pink bars) for the two uranium-treated groups (A_U and B_U). Y-axis: mg uranium per kilogram dry weight (roots) or per kilogram fresh weight (shoots). Error bars = SD (n = 3).

Chlorophyll content, plant morphology, and uranium accumulation in roots and shoots are shown in Figure 1. In original soil (Figure 1a), uranium stress (A_U vs. A) increased chlorophyll a (by 0.18 mg kg^–1^ FW) and chlorophyll b. In sterilized-inoculated soil (Figure 1b), chlorophyll b was similar between B (0.62 mg kg^–1^ FW) and B_U (0.67 mg kg^–1^ FW), whereas chlorophyll a increased by 0.25 mg kg^–1^ FW under uranium stress. Plant morphology (Figure 1c) showed that A_U plants exhibited chlorosis and leaf curling compared to controls, while B_U plants grew vigorously with expanded, dark green leaves. Group B plants also grew well but were shorter than group A. Uranium predominantly accumulated in roots with limited shoot translocation (Figure 1d). In A_U, root uranium content was 8.91 mg kg^–1^ FW higher than that in shoots. In B_U, root uranium was 23.43 mg kg^–1^ FW and shoot uranium was 8.20 mg kg^–1^ FW. Root U was slightly higher in B_U than in A_U, while shoot uranium showed no significant difference between the two groups.

### 3.2 Microbial community diversity and structural characteristics

**FIGURE 2:**
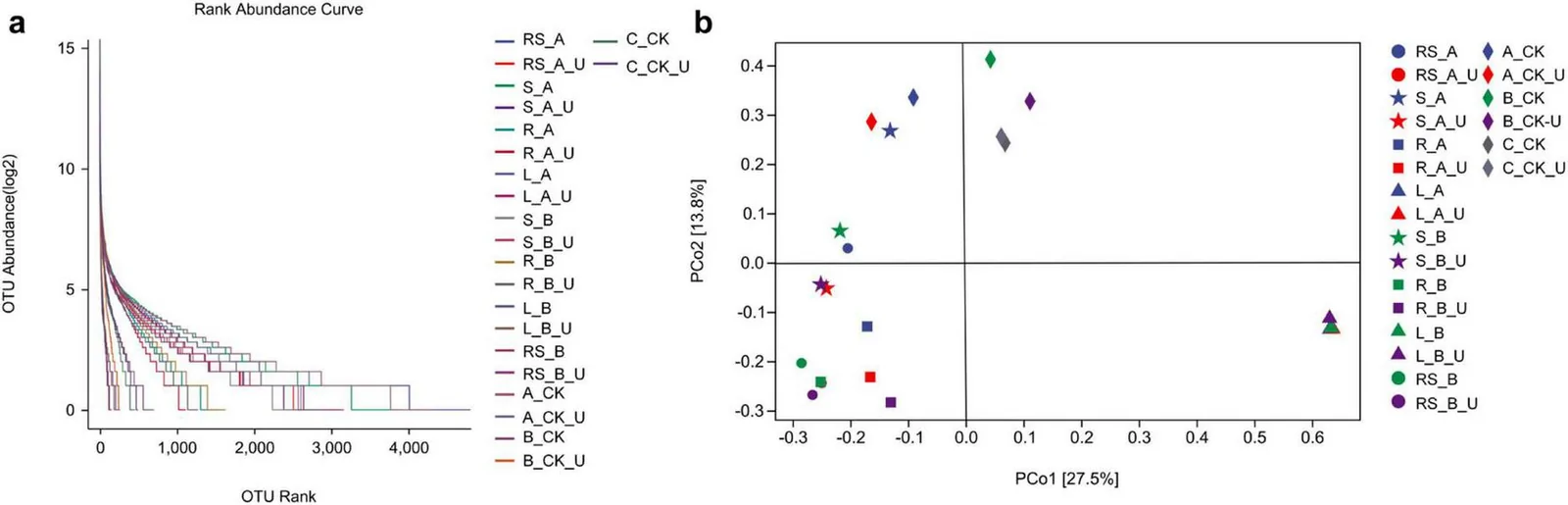
Microbial community diversity and structure across four ecological niches. **(a)** Rank-abundance curves. Each curve shows the relationship between bacterial OTU rank (x-axis, from most to least abundant) and its relative abundance (y-axis, log scale). Wider curves and gentler slopes indicate higher species richness and evenness, Data are from all treatment groups combined (original, reinoculated, sterilized). **(b)** Principal coordinate analysis (PCoA) at the genus level. In the plot, “●” represents rhizosphere soil; “★” represents bulk soil; “■” represents roots; “▲” represents leaves. Colors indicate treatment groups: blue for original soil, red for original soil + U, green for sterilized-inoculated soil, purple for sterilized-inoculated soil + U, and gray for the control sterilized soil. RS_A, rhizosphere soil-original soil treatment; RS_A_U, rhizosphere soil-original soil treatment with uranium addition; RS_B, rhizosphere soil-sterilized-inoculated soil treatment; RS_B_U, rhizosphere soil-sterilized-inoculated soil treatment with uranium addition; S_A, bulk soil-original soil treatment; S_A_U, bulk soil-original soil treatment with uranium addition; S_B, bulk soil-sterilized-inoculated soil treatment; S_B_U, bulk soil-sterilized-inoculated soil treatment with uranium addition; R_A, root-original soil treatment; R_A_U, root-original soil treatment with uranium addition; R_B, root-sterilized-inoculated soil treatment; R_B_U, root-sterilized-inoculated soil treatment with uranium addition; L_A, leaf-original soil treatment; L_A_U, leaf-original soil treatment with uranium addition; L_B, leaf-sterilized-inoculated soil treatment; L_B_U, leaf-sterilized-inoculated soil treatment with uranium addition; A_CK, original soil control; A_CK_U, original soil control with uranium addition; B_CK, sterilized-inoculated soil control; B_CK_U, sterilized-inoculated soil control with uranium addition; C_CK, sterilized soil control; C_CK_U, sterilized soil control with uranium addition.

Rank-abundance curves (Figure 2a) showed that bulk soil and rhizosphere soil samples had the widest horizontal span and gentlest slopes, indicating higher species richness and evenness, while root and leaf samples had narrower spans and steeper slopes. No significant differences in curve morphology were observed among treatment groups within the same ecological niche.

principal coordinate analysis (Figure 2b) revealed clear separation of ecological niches along the first axis: root samples clustered in the third quadrant, rhizosphere and bulk soil across the first and third quadrants, and leaf samples in the fourth quadrant. Along the second axis, rhizosphere and bulk soil showed partial separation with considerable overlap. Within the same niche, samples from different soil treatments (original, sterilized-inoculated, sterilized) and uranium stress mixed without clear clustering. Sterilized soil controls (gray) were distributed at the periphery of the soil cluster, away from original and sterilized-inoculated samples.

The Venn diagram (Supplementary Figure 1) showed limited shared OTUs among treatment groups, with each group harboring many unique taxa, consistent with the partial separation observed in PCoA.

### 3.3 Microbial community composition analysis

Species composition heatmap (Supplementary Figure 2) showed distinct clustering by ecological niche: bulk soil and rhizosphere clustered together, roots formed a separate cluster, and leaves were the most distant, confirming niche as the primary driver. Within the same niche, samples from different treatments showed sub-clustering. Notably, *Massilia* increased in sterilized-inoculated bulk soil, *Bacillus* in several uranium-treated groups, and *Paracoccus* in uranium-stressed original rhizosphere.

**FIGURE 3:**
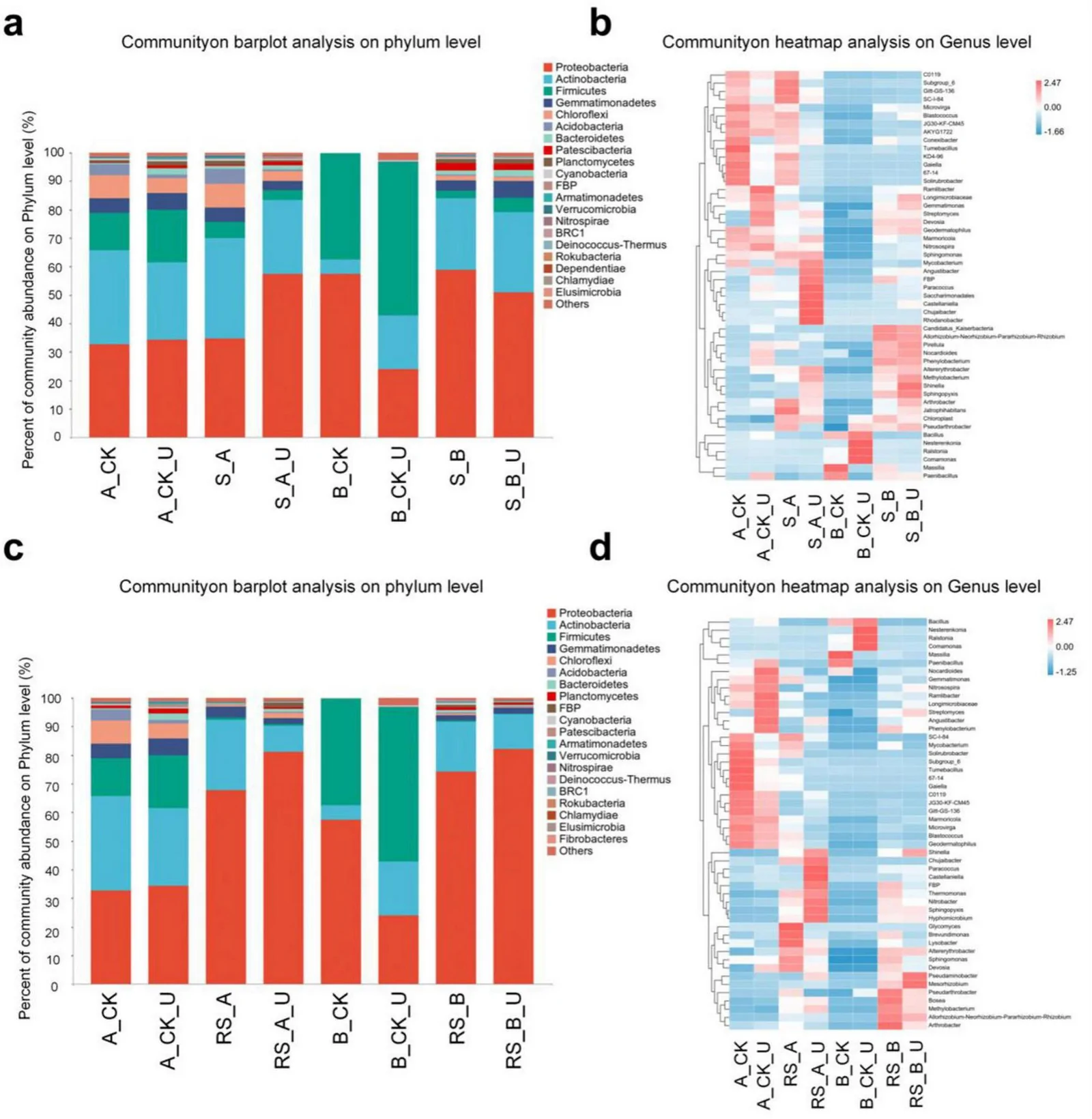
Microbial community composition in bulk and rhizosphere soils. **(a)** Bar plot of the top 20 phyla relative abundance in bulk soil under Original and Sterilized-inoculated treatments. **(b)** Genus-level clustering heatmap of bulk soil communities. **(c)** Bar plot of the top 20 phyla relative abundance in rhizosphere soil under Original and Sterilized-inoculated treatments. **(d)** Genus-level clustering heatmap of rhizosphere soil communities. RS_A, rhizosphere soil-original soil treatment; RS_A_U, rhizosphere soil-original soil treatment with uranium addition; RS_B, rhizosphere soil-sterilized-inoculated soil treatment; RS_B_U, rhizosphere soil-sterilized-inoculated soil treatment with uranium addition; S_A, bulk soil-original soil treatment; S_A_U, bulk soil-original soil treatment with uranium addition; S_B, bulk soil-sterilized-inoculated soil treatment; S_B_U, bulk soil-sterilized-inoculated soil treatment with uranium addition; A_CK, original soil control; A_CK_U, original soil control with uranium addition; B_CK, sterilized-inoculated soil control; B_CK_U, sterilized-inoculated soil control with uranium addition.

Phylum-level analysis (Figures 3a,c) showed that Proteobacteria dominated all soil samples, followed by Actinobacteria. In rhizosphere, sterilized-inoculated treatment increased Proteobacteria and decreased Actinobacteria compared to original soil. Genus-level heatmaps (Figures 3b,d) revealed clear separation between original and sterilized-inoculated soils. In original bulk soil, *Arthrobacter* and *Jatrophihabitans* dominated; under U stress, *Castellaniella*, *Chujaibacter*, and *Rhodanobacter* increased. Sterilized-inoculated bulk soil enriched *Massilia* and the *Allorhizobium* complex. In original rhizosphere, *Lysobacter* and *Brevundimonas* decreased under U stress, while *Chujaibacter*, *Paracoccus*, and *Castellaniella* increased. Sterilized-inoculated rhizosphere enriched *Pseudarthrobacter* and *Bosea*; under uranium stress, *Pseudaminobacter* and *Mesorhizobium* increased.

**FIGURE 4:**
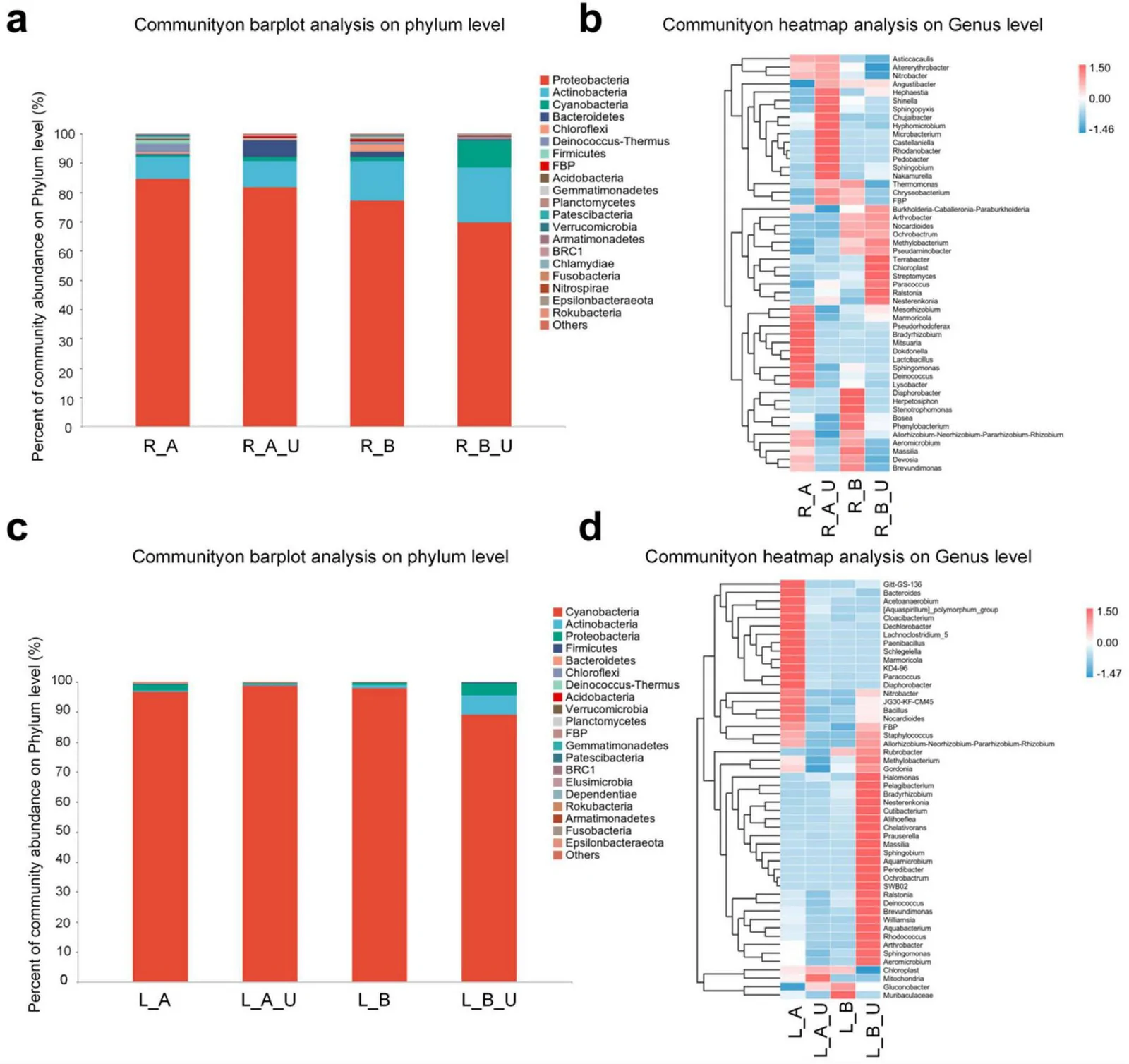
Microbial community composition in roots and leaves. **(a)** Bar plot of the top 20 phyla relative abundance in root under Original and Sterilized-inoculated treatments. **(b)** Genus-level clustering heatmap of roots communities. **(c)** Bar plot of the top 20 phyla relative abundance in leaves under Original and Sterilized-inoculated treatments. **(d)** Genus-level clustering heatmap of leaves communities. R_A, root-original soil treatment; R_A_U, root-original soil treatment with uranium addition; R_B, root-sterilized-inoculated soil treatment; R_B_U, root-sterilized-inoculated soil treatment with uranium addition; L_A, leaf-original soil treatment; L_A_U, leaf-original soil treatment with uranium addition; L_B, leaf-sterilized-inoculated soil treatment; L_B_U, leaf-sterilized-inoculated soil treatment with uranium addition.

In roots (Figures 4a,b), dominant phyla were Proteobacteria, Actinobacteria, Cyanobacteria, and Bacteroidetes. Uranium stress in original soil increased *Castellaniella* and *Pedobacter* but decreased *Mesorhizobium*. In sterilized-inoculated roots, uranium stress increased *Streptomyces* and *Paracoccus*. In leaves (Figures 4c,d), Cyanobacteria dominated; uranium stress decreased Cyanobacteria and increased Actinobacteria. Sterilized-inoculated leaves showed higher baseline abundances of *Gluconobacter*, and unique low-abundance genera (*Chelativorans*, *Massilia*).

Co-occurrence network analysis (Supplementary Figure 3) showed predominantly positive correlations; Proteobacteria and Actinobacteria accounted for most nodes and edges. In the Zi-Pi plot, most nodes had low within- (Zi < 2.5) and among-module (*P*i < 0.62) connectivity; few nodes (Zi ≥ 2.5 or *P*i ≥ 0.62) were also mainly from these two phyla. These results describe co-occurrence patterns, not confirmed ecological interactions.

### 3.4 Differences in microbial composition between original soil and sterilized-inoculated soil

**FIGURE 5:**
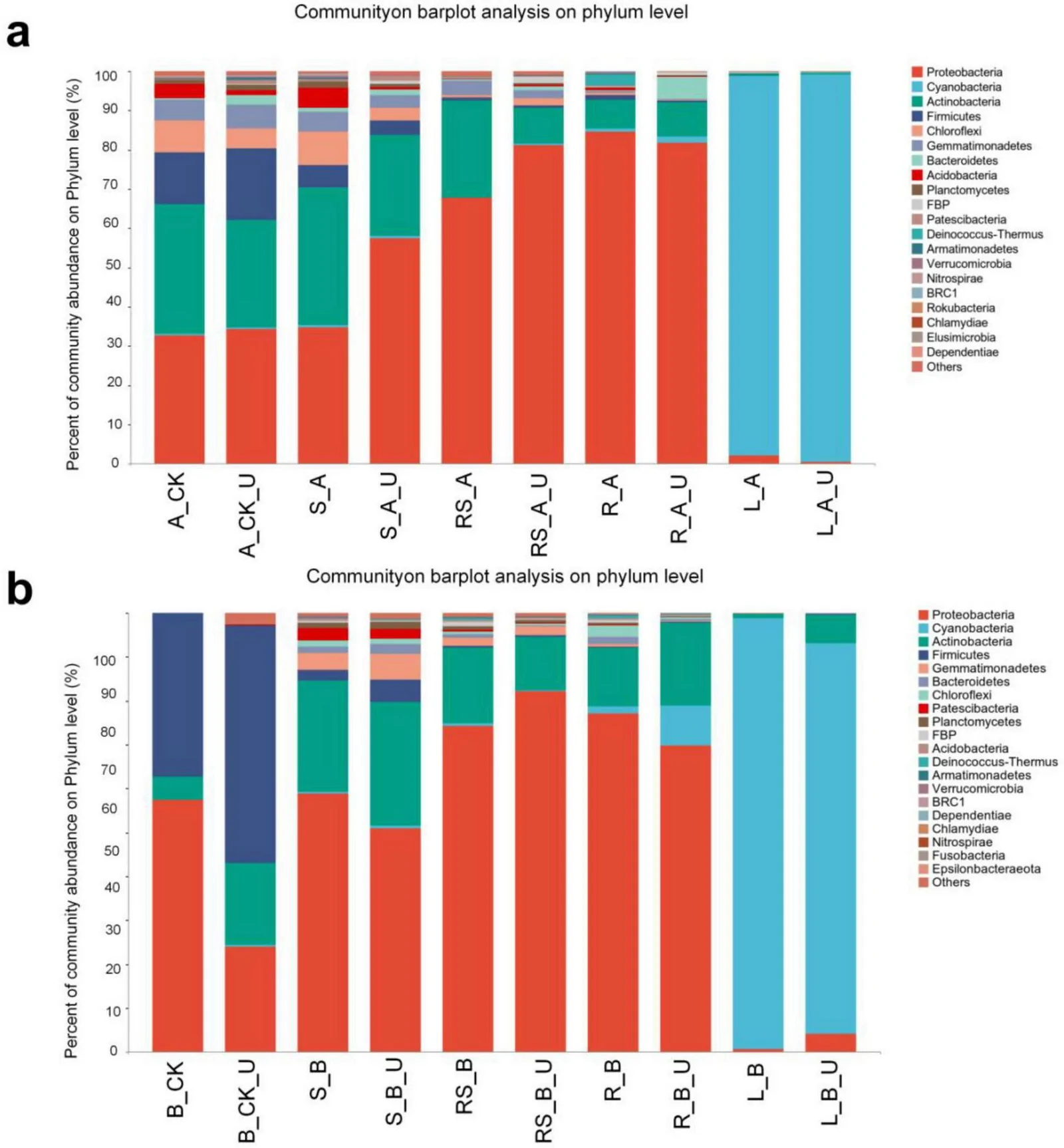
Bar charts showing the relative abundance of the top 20 species at the phylum level in soil. **(a)** Original soil. **(b)** Sterilized-inoculated soil. RS_A, rhizosphere soil-original soil treatment; RS_A_U, rhizosphere soil-original soil treatment with uranium addition; RS_B, rhizosphere soil-sterilized-inoculated soil treatment; RS_B_U, rhizosphere soil-sterilized-inoculated soil treatment with uranium addition; S_A, bulk soil-original soil treatment; S_A_U, bulk soil-original soil treatment with uranium addition; S_B, bulk soil-sterilized-inoculated soil treatment; S_B_U, bulk soil-sterilized-inoculated soil treatment with uranium addition; R_A, root-original soil treatment; R_A_U, root-original soil treatment with uranium addition; R_B, root-sterilized-inoculated soil treatment; R_B_U, root-sterilized-inoculated soil treatment with uranium addition; L_A, leaf-original soil treatment; L_A_U, leaf-original soil treatment with uranium addition; L_B, leaf-sterilized-inoculated soil treatment; L_B_U, leaf-sterilized-inoculated soil treatment with uranium addition; A_CK, original soil control; A_CK_U, original soil control with uranium addition; B_CK, sterilized-inoculated soil control; B_CK_U, sterilized-inoculated soil control with uranium addition.

Phylum-level comparison (Figure 5) revealed significant compositional differences between original and sterilized-inoculated soils. In original soil (Figure 5a), a clear rhizosphere effect was observed: Proteobacteria dominated rhizosphere with higher abundance than in bulk soil, followed by Actinobacteria. Cyanobacteria dominated leaves, while roots, rhizosphere, and unplanted soil were dominated by Proteobacteria, Actinobacteria, and Firmicutes. Chloroflexi and Gemmatimonadetes were mainly confined to soil compartments. In contrast, sterilized-inoculated treatment (Figure 5b) dramatically altered community structure and diminished rhizosphere-bulk differences, leading to homogenization. Proteobacteria remained dominant, but Actinobacteria and Firmicutes increased markedly; Firmicutes even surpassed Proteobacteria in uranium-stressed bulk soil. Oligotrophic phyla such as Acidobacteria were largely replaced.

**FIGURE 6:**
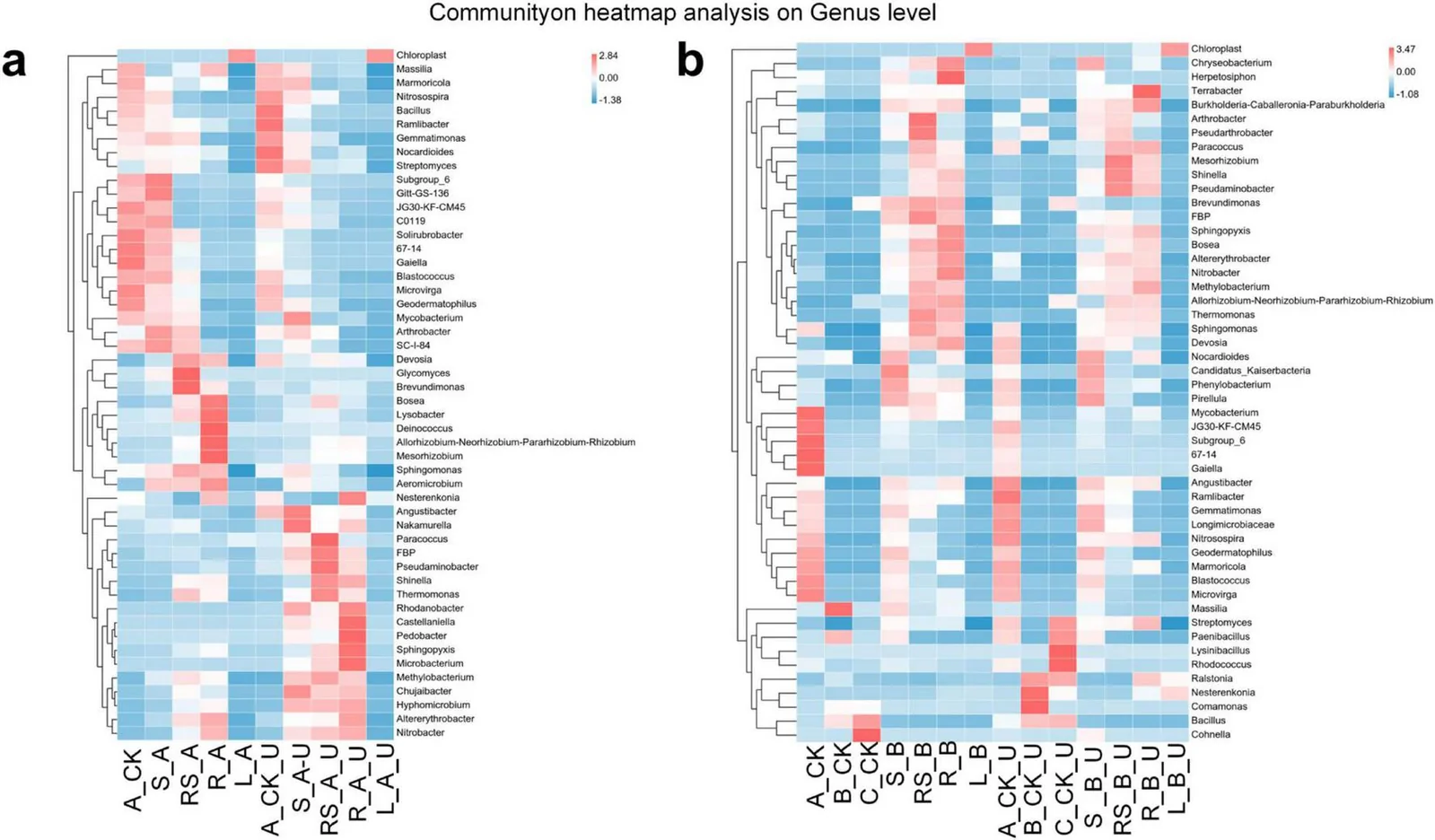
Species clustering heatmap at the genus level. **(a)** Original soil treatment group. **(b)** Sterilized-inoculated soil treatment group. RS_A, rhizosphere soil-original soil treatment; RS_A_U, rhizosphere soil-original soil treatment with uranium addition; RS_B, rhizosphere soil-sterilized-inoculated soil treatment; RS_B_U, rhizosphere soil-sterilized-inoculated soil treatment with uranium addition; S_A, bulk soil-original soil treatment; S_A_U, bulk soil-original soil treatment with uranium addition; S_B, bulk soil-sterilized-inoculated soil treatment; S_B_U, bulk soil-sterilized-inoculated soil treatment with uranium addition; R_A, root-original soil treatment; R_A_U, root-original soil treatment with uranium addition; R_B, root-sterilized-inoculated soil treatment; R_B_U, root-sterilized-inoculated soil treatment with uranium addition; L_A, leaf-original soil treatment; L_A_U: leaf-original soil treatment with uranium addition; L_B, leaf-sterilized-inoculated soil treatment; L_B_U, leaf-sterilized-inoculated soil treatment with uranium addition; A_CK, original soil control; A_CK_U, original soil control with uranium addition; B_CK, sterilized-inoculated soil control; B_CK_U, sterilized-inoculated soil control with uranium addition.

Genus-level heatmaps (Figure 6) further showed that in original soil (Figure 6a), enriched genera included *Massilia*, *Brevundimonas*, and *Lysobacter*. In sterilized-inoculated soil (Figure 6b), clustering boundaries between rhizosphere and bulk blurred, and dominant genera (*Brevundimonas*, *Massilia*, *Streptomyces*) exhibited high abundance in both soil types.

## 4 Discussion

### 4.1 Stress-induced elevation of photosynthetic pigments in rapeseed under uranium stress

Heavy metal stress typically reduces chlorophyll content (Hu et al., 2023), yet here chlorophyll a and b were higher in A_U than in A (Figure 1a) despite visible chlorosis and leaf curling (Figure 1c). This “stress-induced pigment elevation” has also been observed under Cu, Cd, and Pb stress (Zhao et al., 2021). Uranium-triggered ROS attacks chloroplasts and accelerates chlorophyll degradation (Xiong et al., 2023); the higher measured content likely reflects both passive concentration from restricted leaf expansion (Chen et al., 2022) and stress-induced compensatory synthesis that cannot match degradation (Kumar et al., 1996).

In the B_U group, chlorophyll a was highest and leaves were expanded, dark green, and free of chlorosis (Figure 1c), suggesting that the reassembled community alleviated uranium toxicity (Cui et al., 2023). Putative mechanisms include uranium immobilization by enriched genera (*Pseudaminobacter*, *Mesorhizobium*) via phosphatases or EPS (Zhang et al., 2020), antioxidant production (*Streptomyces*) (Rani et al., 2021; Al-Quwaie, 2024), phytohormone/ACC deaminase activity (*Allorhizobium*, *Massilia*) (Wu et al., 2021; Halkuzieva et al., 2024), and siderophore-mediated iron bioavailability (Molnár et al., 2023). However, none of these functions were measured here, so the mechanisms remain speculative. The indigenous community did not show comparable protection, possibly because sensitive taxa like *Lysobacter* declined (Figure 4b), whereas the reassembled community enriched stress-tolerant, putatively growth-promoting taxa; structural integrity does not ensure optimal function (Sarver et al., 2025). In summary, the increase in chlorophyll under uranium stress is a combined outcome of stress response and concentration effects.

### 4.2 Root sequestration of uranium in rapeseed and the limited role of microbial regulation

Uranium mainly accumulated in roots with very low shoot translocation across all treatments (Figure 1d). Efficient root retention is attributed to uranyl complexation with cell wall pectin and cellulose (Malaviya and Singh, 2012). The Casparian strip and suberin lamellae constitute a selective barrier that excludes uranium from the symplastic pathway, leading to vacuolar or cell wall sequestration and limiting xylem loading (Duhan et al., 2023; Sharma et al., 2016). This strategy protects photosynthetic tissues and ensures plant survival (Zheng et al., 2025).

Although sterilized-inoculated treatment altered the soil community, shoot uranium content did not differ significantly between B_U and A_U, and root uranium was only slightly elevated, indicating a limited role for microbial modulation of uranium uptake and translocation (Cui et al., 2023). The plant’s inherent barrier function (e.g., Casparian strip) is genetically controlled and largely unaffected by microbes (Cui et al., 2023; Zheng et al., 2025). Moreover, once uranium is bound to root surfaces, its desorption is slow, and microbial organic acids are inefficient at releasing immobilized uranium in neutral pH soils (Francis and Dodge, 2008). Although *Mesorhizobium* and *Pseudaminobacter* increased under uranium stress, known U-uptake-promoting bacteria (*Pseudomonas*, *Bacillus*) did not enrich, suggesting that the treatment did not select for taxa that efficiently promote uranium translocation. In conclusion, under moderate uranium stress, plant intrinsic tolerance dominates, and microbes indirectly support plant health rather than direct regulation of uranium uptake and distribution.

### 4.3 Primary drivers of microbial community structure: ecological niche overrides soil treatment and uranium stress

Microbial community assembly is governed by habitat filtering, dispersal limitation, species interactions, and stochastic drift (Custer et al., 2022). Bulk soil is carbon-limited (Li et al., 2023), whereas rhizosphere soil receives continuous root exudates, becoming a carbon-rich hotspot (Vives-Peris et al., 2020). Rank-abundance curves and PCoA confirmed niche-specific selection (Figure 2a). Transition into roots imposes physical and immune barriers (Verma et al., 2021; Manoharan et al., 2023), leading to lower diversity and dominance of biofilm-forming, antioxidant-equipped genera like *Mesorhizobium*, *Marmoricola*, and *Sphingomonas* (Ramírez-Larrota and Eckhard, 2022).

The leaf phyllosphere is an extreme habitat with water scarcity, limited nutrients, UV radiation, and high oxygen (Manikandan et al., 2024). Leaf samples formed the most distinct branch in PCoA, dominated by Cyanobacteria (photoautotrophic advantage) and Actinobacteria (stress-resistant spores, polymer degradation) (Wang et al., 2021a; Narsing Rao et al., 2022). Leaves also showed the steepest rank-abundance curves, indicating the lowest evenness.

Although sterilized-inoculated treatment altered initial community composition, partly due to sterilization-induced nutrient release (Berns et al., 2008), samples from the same ecological niche across different soil treatments did not separate clearly in PCoA. Niche filtering outweighs historical effects, and communities converge under identical environmental conditions (Philippot et al., 2024; Ives et al., 2003). The host selection effect (Xiong et al., 2021) overrides soil background. Uranium stress did not markedly restructure communities, possibly because bioavailable uranium remained below a toxic threshold (Bone et al., 2017; Jiao et al., 2023), and enrichment of tolerant taxa (*Castellaniella*, *Rhodanobacter*) buffered community-level effects (Figure 4b).

Community restoration requires propagules and time-dependent niche re-establishment (Du et al., 2025), but structural differences do not always translate into functional differences; functional redundancy is common (Louca et al., 2018). The sterilized-inoculated community differed from original soil (e.g., increased Proteobacteria, decreased Actinobacteria; Figure 4a), yet still supported plant growth under uranium stress.

### 4.4 Community restoration effects of the sterilized-inoculated treatment

Principal coordinate analysis (Figure 2b) showed that sterilized soil controls separated distinctly from original and sterilized-inoculated soils; sterilized-inoculated samples positioned between them but did not fully overlap with original soil. Sterilization-induced alteration is irreversible: even with the same inoculum, final composition cannot perfectly replicate the original state (Delmont et al., 2014). Early colonizers influence succession via resource preemption (Edmonds et al., 2020). In sterilized-inoculated soil, Proteobacteria and Firmicutes increased, while oligotrophic Acidobacteria were largely replaced, consistent with r-strategist dominance and spore-forming persistence (Peng et al., 2022; Liu et al., 2025).

The differentiation between rhizosphere and bulk soil markedly diminished under sterilized-inoculated treatment. In original soil, Proteobacteria were much higher in rhizosphere, and genera like *Massilia*, *Marmoricola*, and *Lysobacter* were enriched (Figure 6a). In contrast, clustering boundaries blurred, and dominant genera (e.g., *Brevundimonas*, *Sphingomonas*) were abundant in both compartments (Figure 6b). Possible explanations include the short plant growth period limiting rhizosphere selection, and sterilization-induced release of labile C/N making the matrix nutrient-rich, thereby reducing the relative importance of root exudates as a carbon source (Condron et al., 2010). In early succession, community composition is more strongly influenced by dispersal and stochastic colonization than by environmental filtering (Wang et al., 2023). Under such conditions, community assembly is governed more by total resource availability than by rhizosphere gradients.

Although sterilized-inoculated soil did not fully restore the original community and weakened rhizosphere-bulk differentiation, rapeseed growth under uranium stress was not inferior; leaf phenotype was even superior (Figure 1). This supports that functional output is not linearly correlated with structural composition, and functional redundancy/compensation exists (Louca et al., 2018). Sterilized-inoculated soil was enriched with putatively multifunctional plant growth-promoting bacteria (*Streptomyces*, *Massilia*, *Pseudarthrobacter*) (Figures 4b, 6b), which have been reported to produce phytohormones, ACC deaminase, siderophores, and induce systemic resistance (Wang et al., 2024). These functions may be underutilized in original soil due to lower abundance of certain functional groups. Sterilization followed by inoculation resets the community, allowing functional taxa to colonize without competition and form a simplified, growth-promoting community.

### 4.5 Selective effects of uranium stress on key microbial taxa

Heavy metal stress exerts differential selective pressures: sensitive taxa decline, while tolerant or functionally complementary taxa become enriched (Ma et al., 2022). *Lysobacter* was highly sensitive to uranium stress, declining in both original and sterilized-inoculated rhizosphere soils (Figure 4b), consistent with reports of membrane disruption and insufficient ROS scavenging (Chen et al., 2020; Song et al., 2024). Its decline could weaken biocontrol (Ayaz et al., 2023), but no disease symptoms were observed, likely because other biocontrol bacteria (*Bacillus*, *Streptomyces*, *Pseudomonas*) increased or remained stable, providing functional compensation (Figures 3b, 4b; Butt et al., 2024). In contrast, *Mesorhizobium*, *Castellaniella*, and *Chujaibacter* increased under uranium stress across multiple niches. *Mesorhizobium* was significantly enriched in sterilized-inoculated rhizosphere and roots (Figure 4b); atypical strains possess PGP traits (IAA, phosphate solubilization, siderophores, ACC deaminase) and can immobilize uranium via surface adsorption or phosphate precipitation (Koli, 2019; Kumar et al., 2023). The higher root uranium in B_U vs. A_U (Figure 1d), without increased shoot uranium, is consistent with *Mesorhizobium*-mediated rhizosphere immobilization rather than enhanced translocation. *Castellaniella*, a denitrifying genus, also increased under uranium stress. Denitrification enzymes can reduce redox-sensitive metals including uranium (Wang et al., 2021b), and certain strains reduce uranyl ions (Tan et al., 2025). Its enrichment thus suggests an active role in converting soluble U(VI) to insoluble U(IV), reducing toxicity (Cui et al., 2023).

Uranium stress also affected leaf phyllosphere: Cyanobacteria decreased while Actinobacteria increased (Figure 3a). Cyanobacteria are sensitive to U-induced photo-oxidative stress (Tiwari et al., 2022), whereas Actinobacteria (e.g., *Nocardioides*, *Streptomyces*, *Arthrobacter*) are adapted to leaf surface extremes and possess antioxidant systems (Shivlata and Satyanarayana, 2017). Their enrichment is consistent with counteracting U-induced oxidative damage. Co-occurrence network analysis showed that Proteobacteria (including *Mesorhizobium*, *Castellaniella*, *Chujaibacter*) and Actinobacteria (including *Streptomyces*, *Nocardioides*) had the highest node counts and central positions, indicating high connectivity. However, these patterns do not demonstrate causal effects, and causal inference is not possible from these data.

**FIGURE 7:**
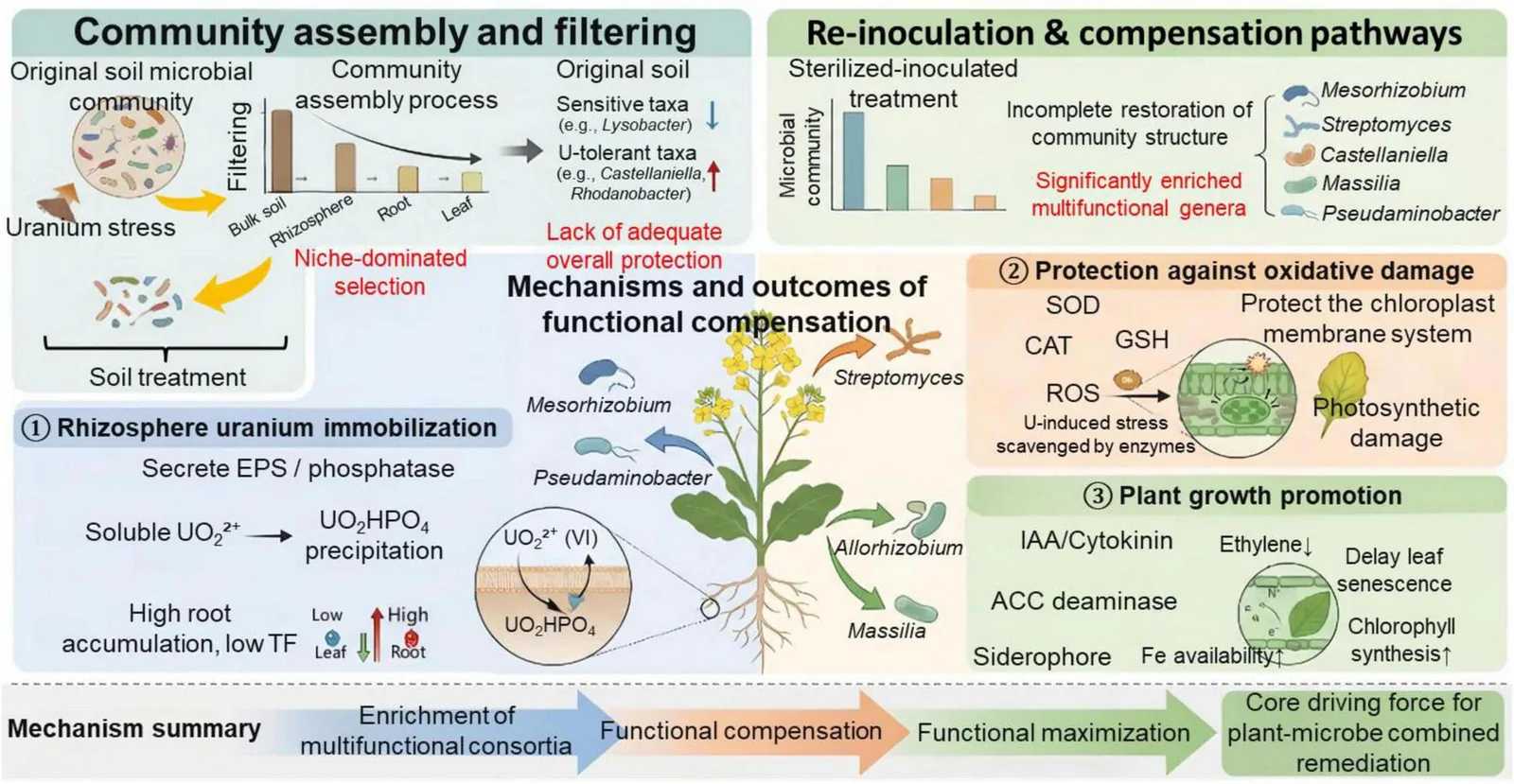
Proposed functional compensation mechanism of the sterilized-inoculated soil microbial community under uranium stress. EPS, extracellular polymeric substances; TF, translocation factor; SOD, superoxide dismutase; CAT, catalase; GSH, glutathione; ROS, reactive oxygen species; IAA, indole-3-acetic acid. Arrows indicate putative pathways inferred from genus-level enrichment and literature; direct experimental validation is required.

Together, these findings support a mechanistic model (Figure 7) in which niche-driven reassembly enriches U-tolerant, growth-promoting taxa (*Mesorhizobium*, *Streptomyces*, *Castellaniella*) that alleviate photosynthetic damage through uranium immobilization, ROS scavenging, and hormonal regulation. Functional compensation across ecological niches does not require complete structural restoration. We did not characterize key soil parameters or porewater uranium fractionation, limiting our ability to separate abiotic from biotic immobilization. However, this does not affect our core conclusion, drawn from comparative microbial and physiological data. Future work should incorporate porewater sampling and X-ray absorption spectroscopy to resolve uranium partitioning and speciation.

## 5 Conclusion

Under uranium stress, rapeseed exhibited increased chlorophyll content yet developed chlorosis and leaf curling, with uranium largely restricted to roots, reflecting an inherent root barrier that limits translocation to shoots. Microbial community structure was driven primarily by ecological niche (bulk soil, rhizosphere, root, leaf) rather than by soil treatment or uranium stress, indicating strong host selection across compartments. The sterilized-inoculated treatment did not fully restore the original community composition, but it was enriched in putatively beneficial genera (e.g., *Mesorhizobium*, *Streptomyces*, *Castellaniella*) and was associated with improved plant phenotype, including alleviated chlorosis and enhanced photosynthetic pigments. These findings suggest that niche differentiation governs community assembly and that maximizing plant-protective functions does not require complete structural restoration of the native microbiome. Functionally, the reassembled community appeared to compensate for structural deficits through enrichment of stress-tolerant, growth-promoting taxa, supporting the concept of functional redundancy and compensation. Future work should focus on validating the putative mechanisms underlying this functional compensation (e.g., uranium immobilization, ROS scavenging, phytohormone production) and on harnessing naturally enriched multifunctional consortia for microbiome-assisted phytoremediation of uranium-contaminated soils.

## Data Availability

The original contributions presented in the study are publicly available. This data can be found here: “Microbial community assembly in rapeseed under uranium stress: 16S rRNA sequencing across soil-root-leaf niches”, with DOI: 10.57760/sciencedb.43243 and CSTR: 31253.11.sciencedb.43243.
