# Correction: Microbial reassembly-driven multi-niche functional compensation alleviates uranium stress in rapeseed

**DOI:** 10.3389/fmicb.2026.1973492

**Published:** 2026-09-03

**Authors:** Frontiers Production Office

**Affiliations:** Frontiers Media SA, Lausanne, Switzerland

**Keywords:** assembly, functional compensation, microbial community, rapeseed, soil sterilization, uranium

The affiliation for Reviewer Shubhransu Nayak was incorrectly stated as “Regional Plant Resource Center, India”. The correct affiliation is “Odisha Biodiversity Board, Odisha, India”.

The original version of this article has been updated.

