# Supplementary material for: Microbial reassembly-driven multi-niche functional compensation alleviates uranium stress in rapeseed
Source: Front Microbiol. 2026 Jul 23;17:1860545. doi: 10.3389/fmicb.2026.1860545 (PMC13446311; doi:10.3389/fmicb.2026.1860545)
Supplement: Supplementary file 1 [file Table_1.docx]

**Table S1** Selected physicochemical properties of the experimental soil.

| Parameter | Value | Unit |
| --- | --- | --- |
| pH | 6.83 | — |
| Organic matter | 13.5 | g·kg⁻¹ |
| Ammonium nitrogen (NH₄⁺-N) | 24.2 | mg·kg⁻¹ |
| Nitrate nitrogen (NO₃⁻-N) | 111.0 | mg·kg⁻¹ |
| Alkaline-hydrolyzable nitrogen | 130.4 | mg·kg⁻¹ |
| Total phosphorus | 1.63 | g·kg⁻¹ |
| Available phosphorus | 599.6 | mg·kg⁻¹ |
| Total potassium | 21.1 | g·kg⁻¹ |
| Available potassium | 165.0 | mg·kg⁻¹ |

**Table S2** Summary of treatment group codes and descriptions.

| Code | Description |
| --- | --- |
| RS_A | Rhizosphere soil – Original soil treatment |
| RS_A_U | Rhizosphere soil – Original soil treatment + U |
| RS_B | Rhizosphere soil – Sterilized-inoculated soil treatment |
| RS_B_U | Rhizosphere soil – Sterilized-inoculated soil treatment + U |
| S_A | Bulk soil – Original soil treatment |
| S_A_U | Bulk soil – Original soil treatment + U |
| S_B | Bulk soil – Sterilized-inoculated soil treatment |
| S_B_U | Bulk soil – Sterilized-inoculated soil treatment + U |
| R_A | Root – Original soil treatment |
| R_A_U | Root – Original soil treatment + U |
| R_B | Root – Sterilized-inoculated soil treatment |
| R_B_U | Root – Sterilized-inoculated soil treatment + U |
| L_A | Leaf – Original soil treatment |
| L_A_U | Leaf – Original soil treatment + U |
| L_B | Leaf – Sterilized-inoculated soil treatment |
| L_B_U | Leaf – Sterilized-inoculated soil treatment + U |
| A_CK | Original soil control (no plants) |
| A_CK_U | Original soil control + U (no plants) |
| B_CK | Sterilized-inoculated soil control (no plants) |
| B_CK_U | Sterilized-inoculated soil control + U (no plants) |
| C_CK | Sterilized soil control (no plants) |
| C_CK_U | Sterilized soil control + U (no plants) |

**Fig. S1** OTU Venn diagram. Each colored block represents a group, and overlapping areas between blocks indicate OTUs shared by the corresponding groups. The number in each segment indicates the count of OTUs contained within that segment. RS_A: rhizosphere soil-original soil treatment; RS_A_U: rhizosphere soil-original soil treatment with uranium addition; RS_B: rhizosphere soil-sterilized-inoculated soil treatment; RS_B_U: rhizosphere soil-sterilized-inoculated soil treatment with uranium addition; S_A: bulk soil-original soil treatment; S_A_U: bulk soil-original soil treatment with uranium addition; S_B: bulk soil-sterilized-inoculated soil treatment; S_B_U: bulk soil-sterilized-inoculated soil treatment with uranium addition; R_A: root-original soil treatment; R_A_U: root-original soil treatment with uranium addition; R_B: root-sterilized-inoculated soil treatment; R_B_U: root-sterilized-inoculated soil treatment with uranium addition; L_A: leaf-original soil treatment; L_A_U: leaf-original soil treatment with uranium addition; L_B: leaf-sterilized-inoculated soil treatment; L_B_U: leaf-sterilized-inoculated soil treatment with uranium addition; A_CK: original soil control; A_CK_U: original soil control with uranium addition; B_CK: sterilized-inoculated soil control; B_CK_U: sterilized-inoculated soil control with uranium addition; C_CK: sterilized soil control; C_CK_U: sterilized soil control with uranium addition.

**Fig. S2** Heatmap of microbial species composition across different ecological niches. RS_A: rhizosphere soil-original soil treatment; RS_A_U: rhizosphere soil-original soil treatment with uranium addition; RS_B: rhizosphere soil-sterilized-inoculated soil treatment; RS_B_U: rhizosphere soil-sterilized-inoculated soil treatment with uranium addition; S_A: bulk soil-original soil treatment; S_A_U: bulk soil-original soil treatment with uranium addition; S_B: bulk soil-sterilized-inoculated soil treatment; S_B_U: bulk soil-sterilized-inoculated soil treatment with uranium addition; R_A: root-original soil treatment; R_A_U: root-original soil treatment with uranium addition; R_B: root-sterilized-inoculated soil treatment; R_B_U: root-sterilized-inoculated soil treatment with uranium addition; L_A: leaf-original soil treatment; L_A_U: leaf-original soil treatment with uranium addition; L_B: leaf-sterilized-inoculated soil treatment; L_B_U: leaf-sterilized-inoculated soil treatment with uranium addition; A_CK: original soil control; A_CK_U: original soil control with uranium addition; B_CK: sterilized-inoculated soil control; B_CK_U: sterilized-inoculated soil control with uranium addition; C_CK: sterilized soil control; C_CK_U: sterilized soil control with uranium addition.

**Fig. S3** Microbial association analysis. **a**, Co-occurrence network diagram. Nodes represent OTUs in the samples, with node size proportional to abundance (in units of log2(CPM/n)). **b**, Zi-Pi plot. The X- and Y-axes represent the Pi and Zi values of nodes within the network, respectively. Node size is proportional to abundance (in units of log2(CPM/n)).
